# Methylene blue alleviates nuclear and mitochondrial abnormalities in progeria

**DOI:** 10.1111/acel.12434

**Published:** 2015-12-14

**Authors:** Zheng‐Mei Xiong, Ji Young Choi, Kun Wang, Haoyue Zhang, Zeshan Tariq, Di Wu, Eunae Ko, Christina LaDana, Hiromi Sesaki, Kan Cao

**Affiliations:** ^1^ Department of Cell Biology and Molecular GeneticsUniversity of MarylandCollege ParkMD20742USA; ^2^ Center for Bioinformatics and Computational BiologyUniversity of MarylandCollege ParkMD20742USA; ^3^ Department of Cell BiologyJohns Hopkins University School of MedicineBaltimoreMD21205USA

**Keywords:** aging, methylene blue, mitochondria, PGC1‐α, progeria

## Abstract

Hutchinson–Gilford progeria syndrome (HGPS), a fatal premature aging disease, is caused by a single‐nucleotide mutation in the *LMNA* gene. Previous reports have focused on nuclear phenotypes in HGPS cells, yet the potential contribution of the mitochondria, a key player in normal aging, remains unclear. Using high‐resolution microscopy analysis, we demonstrated a significantly increased fraction of swollen and fragmented mitochondria and a marked reduction in mitochondrial mobility in HGPS fibroblast cells. Notably, the expression of PGC‐1α, a central regulator of mitochondrial biogenesis, was inhibited by progerin. To rescue mitochondrial defects, we treated HGPS cells with a mitochondrial‐targeting antioxidant methylene blue (MB). Our analysis indicated that MB treatment not only alleviated the mitochondrial defects but also rescued the hallmark nuclear abnormalities in HGPS cells. Additional analysis suggested that MB treatment released progerin from the nuclear membrane, rescued perinuclear heterochromatin loss and corrected misregulated gene expression in HGPS cells. Together, these results demonstrate a role of mitochondrial dysfunction in developing the premature aging phenotypes in HGPS cells and suggest MB as a promising therapeutic approach for HGPS.

## Introduction

Hutchinson–Gilford progeria syndrome (HGPS) is a rare, autosomal dominant disorder characterized by rapid, premature aging in children (Eriksson *et al*., 2003; Capell & Collins, 2006; Gordon *et al*., 2007, 2012). It is caused by a C to T mutation in the 11th exon of the *LMNA* gene (1824C>T) which leaves the amino acid code unchanged, instead activating a cryptic splice site. When used, this splice site removes the last 150 nucleotides from the 11th exon, resulting in an internal 50 amino acid deletion in the lamin A protein (De Sandre‐Giovannoli *et al*., 2003; Eriksson *et al*., 2003). This deletion interferes with posttranslational processing by removing a key protease cleavage site, leading to permanent farnesylation and aberrant anchorage of the mutant lamin A, termed progerin, to the nuclear membrane (Eriksson *et al*., 2003; Capell & Collins, 2006). The abnormal presence of progerin disrupts the integrity of the nucleoskeleton, causing high levels of nuclear abnormalities including nuclear blebbing, altered chromatin organization, transcriptional changes, and aberrant mitosis (Goldman *et al*., 2002, 2004; Columbaro *et al*., 2005; Liu *et al*., 2005; Shumaker *et al*., 2006; Cao *et al*., 2007; Dechat *et al*., 2007, 2008; McCord *et al*., 2013; Stancheva & Schirmer, 2014).

Mitochondria are complex organelles that are believed to play a significant role in biological aging (Shigenaga *et al*., 1994; Bratic & Larsson, 2013). They form a sophisticated, dynamic, and tubular network that moves along microtubules and actin fibers (Morris & Hollenbeck, 1995; Nunnari & Suomalainen, 2012). Mitochondria undergo a delicate balance between fusion and fission to maintain a functional population, where dysfunctional mitochondria are destroyed via autophagy and new mitochondria are made to replace them (Boldogh & Pon, 2007; Cerveny *et al*., 2007). Dysfunctional mitochondria can cause systemic problems, increasing amounts of reactive oxygen species (ROS) and triggering DNA and protein damage (Kirkinezos & Moraes, 2001).

Elevated ROS has been reported in HGPS cells (Viteri *et al*., 2010; Lattanzi *et al*., 2012). In addition, a marked downregulation of mitochondrial oxidative phosphorylation proteins, reduced ATP levels, and mitochondrial dysfunction were reported in HGPS fibroblasts as well as in HGPS mouse models (Rivera‐Torres *et al*., 2013; Villa‐Bellosta *et al*., 2013). This evidence suggests potential mitochondrial defects in HGPS. However, it remains unclear to what extent mitochondrial dysfunction contributes to the premature aging phenotypes associated with HGPS. In this study, we characterized HGPS‐specific mitochondrial defects and studied the mechanisms underlying these abnormalities. Moreover, we tested MB, an antioxidant compound known to stimulate mitochondrial function. Our results show that MB treatment improves not only the mitochondrial morphology and function but also seems to specifically rescue the premature aging phenotypes in HGPS nuclei. Our study points to a promising new treatment for HGPS.

## Results

### Progerin induces swollen and fragmented mitochondria and inhibits mitochondrial mobility

**Figure 1:**
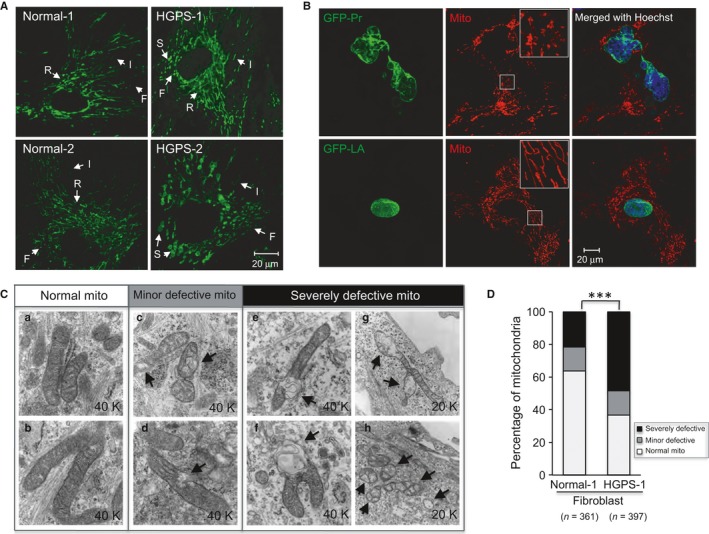
Morphological abnormalities of mitochondria in HGPS fibroblasts. (A) Representative fluorescence images of the mitochondria stained by MitoTracker Green FM in two normal fibroblast lines and two HGPS fibroblast lines. R: reticular mitochondria; I: intermediate mitochondria; F: fragmented mitochondria; S: swollen mitochondria. Scale bar, 20 μm. (B) Representative fluorescence images of normal fibroblasts transduced with lentivirus expressing either GFP‐lamin A (GFP‐LA) or GFP‐progerin (GFP‐Pr). The mitochondria were stained by MitoTracker Red CMXRos. Scale bar, 20 μm. (C) Representative transmission electron micrographs (TEM) of mitochondria taken from either normal‐1 (control) or HGPS‐1 (G608G) fibroblasts showing various morphological alterations ranging from ‘normal mitochondria’ (a‐b), ‘minor defective mitochondria’ (c‐d) to ‘severe defective mitochondria’ (e‐h). These three general categories for phenotype grading were classified based on the intactness of membrane (outer, inner and cristae), matrix integrity and overall organelle shape: mitochondria with intact membrane and matrix were considered as ‘normal mitochondria (a‐b); mitochondria with broken membrane or with small vacuole areas in matrix (<20% of the total area) were considered as ‘minor defective mitochondria’ (c‐d); and mitochondria that were either morphologically abnormal (swollen or budding) or with large vacuole areas (over 20% of the total area) were defined as ‘severe defective mitochondria’ (e‐h). Over 300 mitochondria in either normal‐1 or HGPS‐1, fibroblasts were blindly scored according to these criteria. Arrows pointed to abnormalities. (D) Percentages of mitochondria with different types of abnormalities in normal‐1 or HGPS‐1 fibroblasts. The number of mitochondria that were blindly scored in each group is indicated in the parentheses (***P <* *0.001 by chi‐squared test).

Mitochondria have variable morphologies classified into reticular, intermediate, or fragmented according to their shape and size. This morphological diversity is linked to the mitochondrial function (Capaldi *et al*., 2002; Cerveny *et al*., 2007). To visualize mitochondria in live cells, MitoTracker^**®**^ was added to culture medium to stain mitochondrial membrane. Two HGPS cell lines (HGPS‐1 and 2) and passage‐matched controls (normal‐1 and 2) were analyzed in parallel (cell line information in Table S1). Western blotting analysis verified the expression of progerin in HGPS 1 and 2 cells (Fig. S1A). Interestingly, HGPS‐2 showed a much higher expression of progerin than HGPS‐1 (Fig. S1A). The G1 phase normal‐1 and 2 cells showed the expected perinuclear reticular mitochondrial network with small fractions of intermediate and fragmented mitochondria (Fig. 1A and Fig. S1B–D). In contrast, both HGPS cell lines showed a noticeable increase of fragmented mitochondria, with this phenotype being more severe in HGPS‐2 relative to HGPS‐1 (Fig. 1A and Fig. S1B–D), which is consistent with the progerin‐level differences in these lines. In HGPS‐2, a complete loss of the perinuclear reticular network and obvious mitochondrial swollenness were observed (Fig. 1A).

To directly examine the effect of progerin on mitochondrial morphology, we infected a normal fibroblast cell line (normal‐1) with either GFP‐lamin A (GFP‐LA)‐ or GFP‐progerin (GFP‐Pr)‐expressing lentiviruses. Two weeks after infection, mitochondria in the GFP‐Pr‐expressing cells exhibited a swollen and fragmented phenotype (Fig. 1B) that is similar to those in primary HGPS fibroblasts (Fig. 1A). Next, we applied transmission electron microscopy (TEM) to elucidate these mitochondrial defects at a higher resolution (Fig. 1C). We scored individual mitochondrion according to the severity of its phenotype blindly in normal and HGPS samples (Materials and methods). As vulnerable targets of ROS, mitochondria can display some abnormalities under normal physiological conditions (Chan, 2006). These defects are similar to what we categorized here as ‘minor defects’, which showed similar proportions in both normal and HGPS samples. Notably, there was a significant twofold increase in the category of severely damaged mitochondria in HGPS fibroblasts compared to normal cells (Fig. 1D), implying a potential problem in mitochondrial biogenesis.

**Figure 2:**
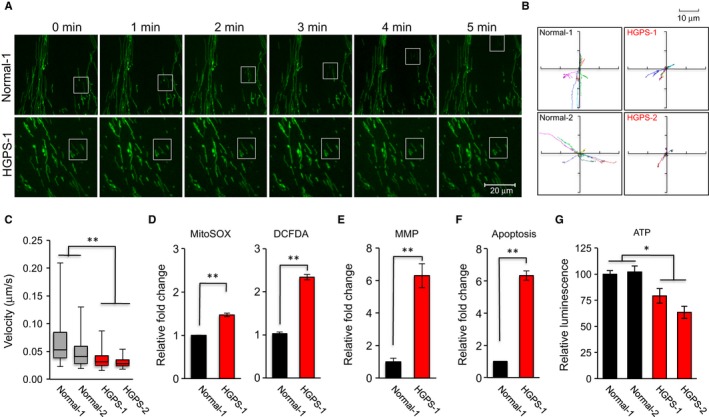
Defective mitochondrial mobility and function in HGPS fibroblasts. (A) Time‐lapse images of mitochondria labeled with MitoTracker Green FM in normal‐1 or HGPS‐1 cells. Images were acquired every 10 s over a total of 5 min time course by a spinning disk confocal microscope. Scale bar, 20 μm. (B) Movement traces of 10 representative mitochondria per each cell line in two normal fibroblast lines and two HGPS fibroblast lines. Different colors represent individual mitochondrion, and each dot shows one acquired time point at 10 s. intervals. (C) Velocity quantification of mitochondrial movement in two cell lines from each of normal and HGPS fibroblasts. Sixty mitochondria per cell line were randomly selected for velocity analysis (**P <* *0.01). (D) Relative fold change of mitochondrial superoxide (MitoSOX, left) and intracellular ROS (DCFDA, right) amounts measured by FACS analysis in normal‐1 and HGPS‐1 fibroblasts (**P <* *0.01). (E & F) Relative cell percentages with disrupted mitochondrial membrane potential (MMP, E) and apoptosis (F) (**P <* *0.01). (G) ATP production in two normal and two HGPS fibroblasts (*P <* *0.05).

To characterize mitochondrial movement in HGPS cells, we conducted live mitochondrial imaging using MitoTracker^**®**^ Green (Fig. 2A and Fig. S2A). The mitochondria in HGPS cells showed significantly shorter travel distances and slower speeds than those in normal cells (Fig. 2B,C). This observation was further verified in normal cells transduced by lentiviruses expressing either GFP‐LA or GFP‐Pr (Fig. S2B,C). Together, these experiments indicate that progerin causes fragmented and swollen mitochondria and inhibits mitochondrial movement.

### Mitochondria in progerin‐expressing cells show impaired function

Previous evidence suggested an elevated level of cellular ROS in HGPS cells (Kimura *et al*., 2007; Viteri *et al*., 2010; Rivera‐Torres *et al*., 2013; Villa‐Bellosta *et al*., 2013). To further analyze the functional defects in HGPS mitochondria, we measured both the mitochondrial‐specific superoxide (MitoSOX) and the intracellular ROS (DCFDA) and found both were significantly higher in progerin‐expressing fibroblast cells compared to control (Fig. 2D and Fig. S2D). Also no detectable change in mtDNA amount was found when mtDNA amounts in HGPS‐1 and his biological mother normal‐2 were compared (Fig. S2E). Moreover, an increase in cells with mitochondrial membrane potential (MMP) disruption and/or apoptosis (Fig. 2E,F, raw data in supplemental Fig. S3A,B) and a reduction in cellular ATP production (Fig. 2G) were also observed in HGPS samples.

Furthermore, to determine whether the progerin‐induced mitochondrial defects are present in other cell types besides fibroblasts, we examined HGPS iPSC‐derived smooth muscle cells (iSMCs) (Zhang *et al*., 2014), in which mitochondrial function is essential. Notably, most of the mitochondria in HGPS iSMCs appear to be round and swollen, and a significantly elevated ROS was also detected in these cells (Fig. S4A,B).

### Progerin suppresses the expression of PGC‐1α

**Figure 3:**
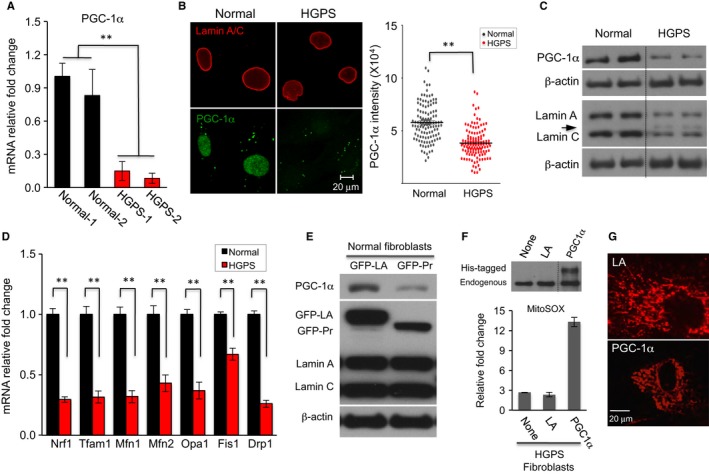
Reduced PGC‐1α expression in HGPS cells. (A) Quantitative RT–PCR analysis of PGC‐1α expression in two normal and two HGPS fibroblast cell lines (**P <* *0.01). (B) Representative immunofluorescence images of normal‐1 and HGPS‐1 fibroblasts stained with antilamin A/C (Red) and anti‐PGC‐1α (Green) antibodies. Scale bar, 20 μm. The PGC‐1α green fluorescence intensity was plotted in the right dot plots. More than 100 nuclei were randomly selected from normal‐1 or HGPS‐1 fibroblasts for intensity analysis (**P <* *0.01). (C) Western blotting analysis with anti‐PGC‐1α, antilamin A/C, or anti‐β‐actin antibodies in normal‐1 and HGPS‐1 fibroblasts (two technical replicates per each fibroblast). Arrow points to progerin protein. The black dotted line indicates that the bands have been spliced together from the same film. (D) Quantitative RT–PCR analysis of Nrf1, Tfam1, Mfn1, Mfn2, Opa1, Fis1, and Drp1 in normal‐1 and HGPS‐1 fibroblasts (**P <* *0.01). (E) Western blot analysis with anti‐PGC‐1α, antilamin A/C, and anti‐β‐actin antibodies in normal fibroblasts transduced with lentivirus expressing GFP‐lamin A (GFP‐LA) or GFP‐progerin (GFP‐Pr). (F) Western blot analysis with anti‐PGC‐1α antibody confirmed the exogenous expression of His‐tagged human PGC‐1α in HGPS‐1 fibroblasts infected with lentivirus containing His‐tagged human PGC‐1α genes for 3 days (upper panel). Lentivirus containing lamin A gene (LA) is used as a control here. The black dotted line indicates that the bands have been spliced together from the same film. The MitoSOX were checked by FACS analysis in the same fibroblast cells (lower panel). (G) Representative cell images of mitochondria in either LA or PGC‐1α lentiviral‐infected HGPS‐1 cells. Scale bar, 20 μm.

PGC‐1α serves as a master inducer of mitochondrial biogenesis through its co‐activation of nuclear respiratory factors (NRFs), which control the expression of nuclear genes encoding mitochondrial proteins (Wu *et al*., 1999; Finck & Kelly, 2006). Using adipogenesis array, we previously reported that in HGPS adipocytes, PGC‐1α was the most severely downregulated gene among the 84 genes involved in energy metabolism (Xiong *et al*., 2013). Thus, to understand how progerin causes mitochondrial defects, we first examined PGC‐1α expression in HGPS fibroblasts. Quantitative RT–PCR experiments revealed that the PGC‐1α mRNA level declined by eight folds in HGPS cell lines compared to normal cell lines (Fig. 3A). Immunofluorescence with an anti‐PGC‐1α antibody showed weakened or a complete loss of PGC‐1α nuclear staining in HGPS cells (Fig. 3B). Western blotting analysis further confirmed the reduction of PGC‐1α protein in HGPS cells (Fig. 3C). Consistent with the downregulation of PGC‐1α, we found that most of PGC‐1α's downstream target genes, including Nrf1, Tfam1, Mfn1, Mfn2, Opa1, Fis1, and Drp1 (Dillon *et al*., 2012), were significantly suppressed in HGPS cells (Fig. 3D). We further confirmed the inhibitory effect of progerin on PGC‐1α using lentiviruses expressing GFP‐progerin (Fig. 3E). To test whether restoration of PGC‐1α in HGPS cells alleviates the mitochondrial defects, lentiviruses carrying either a control lamin A gene (LA) or human PGC‐1α gene were applied to HGPS fibroblast cells (Fig. 3F). To our surprise, we did not detect any obvious improvements in mitochondrial morphology or behavior (representative cell images shown in Fig. 3G). Instead, an elevated mitochondrial ROS in PGC‐1α‐expressing cells was found, in comparison with the control lamin A (LA)‐expressing cells (Fig. 3F). Taken together, these results suggest that while PGC‐1α has an established role in regulating mitochondrial biogenesis, correction of PGC‐1α expression alone might not be sufficient to rescue the severe mitochondrial phenotypes caused by progerin.

### MB promotes cell proliferation and rescues hallmark nuclear blebbing phenotype in HGPS fibroblasts

MB is an effective agent to delay cellular senescence at nanomolar concentration and prevent age‐related decline in cognitive function and grip strength (Atamna *et al*., 2008; Harrison *et al*., 2014). It also exhibits potent antioxidant effects in mitochondria due to its redox property. The cycling between reduced (MBH2) and oxidized (MB) forms facilitates electron transfer, thus preventing electron leakage, increasing mitochondrial oxidative phosphorylation, and reducing ROS overproduction under pathological conditions (Atamna & Kumar, 2010). Based on these previous studies, we examined whether the treatment with MB could alleviate dysfunctional mitochondria and delay premature senescence associated with HGPS cells.

**Figure 4:**
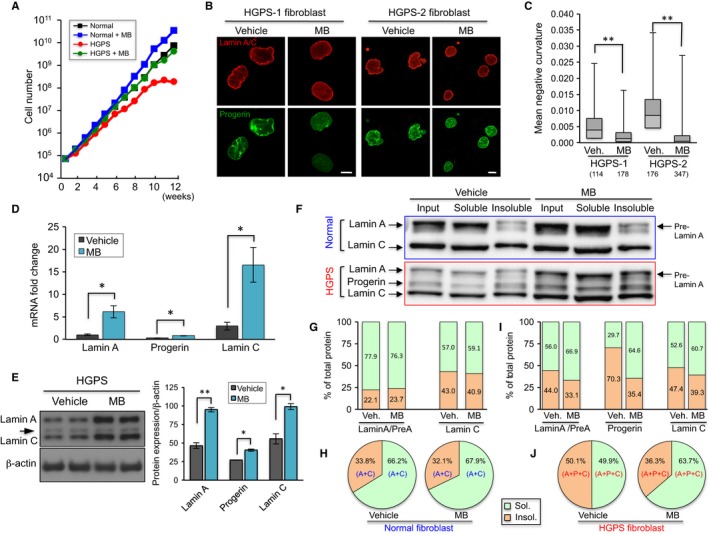
MB improves HGPS nuclear morphology and solubilizes progerin. (A) Growth curves of normal‐1 and HGPS‐1 fibroblasts treated with vehicle or methylene blue (MB) at 100 nm for 12 weeks. The treatment started at the passage 14 for all cell lines and ended at passage 20 when the HGPS‐1 cells in mock treatment reached senescence. (B) Representative immunofluorescence images of HGPS‐1 and HGPS‐2 fibroblasts treated with vehicle or MB 100 nm for 8 weeks. Red: antilamin A/C antibody, Green: antiprogerin antibody. Scale bar, 20 μm. (C) Quantification of mean negative curvature (MNC) in HGPS‐1 and HGPS‐2 fibroblasts treated with vehicle or MB at 100 nm for 8 weeks. Over 100 nuclei from each group were randomly selected, and their boundaries were quantitatively analyzed using the nuclear morphology analysis software that was reported previously (Driscoll *et al*., 2012). The number of nuclei in each group is indicated in the parentheses underneath each cell type (**P <* *0.01). (D) Quantitative RT–PCR analysis of lamin A, progerin and lamin C mRNA levels in HGPS‐1 fibroblasts treated with vehicle or MB at 100 nm for 6 weeks (*P < 0.05). (E) Left: Western blotting analysis with antilamin A/C and anti‐β‐actin antibodies in HGPS‐1 fibroblasts treated with vehicle or MB 100 nm for 6 weeks. Two technical replicates per sample were shown. Right: Quantification of relative protein amounts of lamin A, progerin, and lamin C. (F) Nuclear fractionation and Western blotting showing the input, soluble and insoluble fractions of lamin A, progerin and lamin C. Normal‐1 and HGPS‐1 fibroblasts were in treatment with vehicle or 100 nm MB for 6 weeks. (G) Percentages of soluble (Green) and insoluble (Orange) fractions of lamin A (including prelamin A) and lamin C in normal‐1 cells. Data for each protein were collected based on the band intensities. Sol% = Sol/(Sol + Insol) X 100%; Insol = Insol/(Sol + Insol) X 100%. (H) Pie graphs showing the combined analyses of soluble and insoluble fractions of lamin A and lamin C (A+C) in normal‐1 cells. (I) Percentages of soluble (Green) and insoluble (Orange) fractions of lamin A (including prelamin A), progerin and lamin C in HGPS‐1 cells. Data for each protein were collected based on the band intensities. Sol% = Sol/(Sol + Insol) X 100%; Insol = Insol/(Sol + Insol) X 100%. (H) Pie charts showing the combined analyses of soluble and insoluble fractions of lamin A, progerin, and lamin C (A+P+C) in HGPS‐1 cells.

HGPS and normal fibroblasts at passage 10 were treated with buffer (PBS) or MB at 100 nm for 12 weeks. Similar to the results in a previous study (Atamna *et al*., 2008), MB treatment improved normal cell proliferation (Fig. 4A). The untreated HGPS cells proliferated at a slower rate than the normal cells and stopped dividing after 9 weeks’ culture. Significantly, the MB‐treated HGPS cells continued dividing until the termination of a 12‐week experiment, and their proliferation curve was almost identical to that of the mock‐treated normal cells (Fig. 4A). Consistent with the cell proliferation analysis, both senescence‐associated β‐galactosidase (SA‐β‐gal) assay and p16 expression analysis showed that MB‐treated HGPS cells were younger than the mock‐treated cells (Fig. S5A,B). Nuclear blebbing has been considered the hallmark phenotype in HGPS cells, resulting from the abnormal anchorage of progerin to the inner nuclear membrane (Capell *et al*., 2005, 2008; Dechat *et al*., 2009, 2010). To assay MB's effects on nuclear morphology, mock‐ and MB‐treated cells were immunostained with antilamin A/C and antiprogerin antibodies (Fig. 4B). We found that the mean negative curvature, a direct measure of abnormalities of the nuclear shape (Driscoll *et al*., 2012), was significantly decreased in HGPS cells after MB treatment (Fig. 4C). Thus, we conclude that MB treatment remediates cell proliferation and nuclear morphology defects in HGPS cells.

### MB upregulates A‐type lamins and significantly improves the solubility of progerin

It has been shown that MB is a highly permeable molecule that can easily enter the nucleus and bind to nuclear DNA (Nogueira & Gonzalez, 2014). Thus, we speculated that besides targeting mitochondria, MB might enter and function in the HGPS nucleus, thereby improving the nuclear phenotypes. In an attempt to understand how MB reduced nuclear blebbing and promoted cell proliferation, we compared the amounts of lamins A and C and progerin in mock‐ and MB‐treated HGPS fibroblasts, using quantitative RT–PCR (Fig. 4D) and Western blotting (Fig. 4E). We observed a transcriptional upregulation and a corresponding increase in protein amounts for all three A‐type lamins after MB treatment (Fig. 4D,E). Among them, the amounts of lamins A and C appeared to have a greater increase than progerin (Fig. 4D,E).

To visualize these A‐type lamins in MB‐treated HGPS cells, we conducted confocal microscopy analysis using antibodies against lamin A/C and/or progerin (antilamin A/C antibody recognizes all three and antiprogerin antibody is progerin specific). Interestingly, antiprogerin antibody staining revealed a clear redistribution of progerin from the nuclear rim into the nucleoplasm in MB‐treated HGPS cells (Figs S5C and S6). To further understand this redistribution of progerin in MB treatment, a biochemical nuclear fractionation experiment (Materials and methods) was carried out to separate the membrane‐bounded progerin fraction (Insoluble) from the nucleoplasmic fraction (Soluble) in both HGPS and normal cells (Fig. 4F). Notably in this experiment, the membrane‐bounded prelamin A only presented in the insoluble fractions (Fig. 4F). In normal cells, 75–80% of lamin A and 55–60% lamin C were soluble (Fig. 4F upper panel and 4G). Figure 4H showed a combined analysis of soluble fractions of both lamin A and lamin C (A+C), revealing that in normal fibroblasts, about 65% of A+C are soluble (Fig. 4H). Moreover, we found that MB treatment did not cause a significant change in the solubility of lamin A or C in the normal fibroblasts (Fig. 4G,H).

In a parallel experiment, we examined the solubility of lamins A and C and progerin in HGPS fibroblasts (Fig. 4F‐lower panel and 4I). We noticed the following differences in HGPS cells: first, the quantitative analysis indicated that only about 30% of progerin was soluble in the mock‐treated HGPS sample, which is much lower than either lamin A or lamin C (Fig. 4I‐middle columns). Second, there was a decrease in soluble fraction of lamin A from ~75% in normal cells to ~55% in HGPS sample (Fig. 4I‐left columns), which is likely due to the dimerization or oligomerization of the insoluble progerin with lamin A. Lastly, after MB treatment, the soluble fraction of progerin was increased dramatically from 30% to ~65%, and the soluble fractions of lamins A and C also increased by 8–10% correspondingly (Fig. 4I). The combined analysis of all soluble fractions of A‐type lamins (A+P+C) revealed that MB treatment pumped the solubility of A‐type lamins from 50% to near 64% (Fig. 4J), which became comparable with the percentage in normal cells (~67%, Fig. 4H). Together, these experiments demonstrate that MB treatment promotes the expression of A‐type lamins, especially lamin A and C. In addition, MB treatment released progerin from the nuclear membrane and significantly increased the solubility of progerin.

### MB reduces ROS, improves overall mitochondrial health, and stimulates PGC‐1α expression

**Figure 5:**
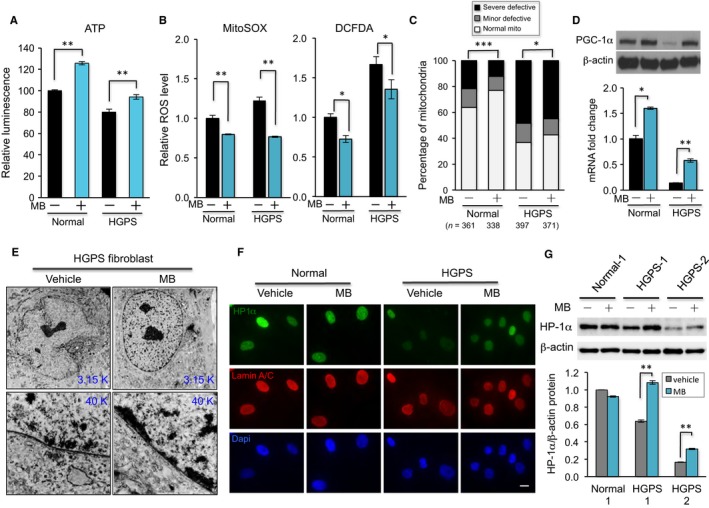
Methylene blue improves mitochondrial function and rescues heterochromatin loss phenotype in HGPS cells. (A) ATP production in normal‐1 and HGPS‐1 fibroblasts treated with vehicle or MB 100 nm for 5 weeks (**P <* *0.01). (B) Relative fold change of mitochondrial superoxide levels (MitoSOX, Left) and intracellular ROS levels (DCFDA, Right) measured by FACS analysis in normal‐1 and HGPS‐1 fibroblasts that were treated with vehicle or 100 nm MB for 8 weeks (*P <* *0.05; **P <* *0.01). (C) Percentages of mitochondria with different levels of abnormalities in normal‐1 and HGPS‐1 fibroblasts that were treated with vehicle or 100 nm MB for 8 weeks. The total number of mitochondria that were blindly scored in each group is indicated in the parentheses (*P <* *0.05; ***P <* *0.001 by chi‐squared test). (D) Upper: Western blot analysis with anti‐PGC‐1α and anti‐β‐actin antibodies in normal‐1 and HGPS‐1 fibroblasts treated with vehicle or 100 nm MB for 8 weeks. Lower: Quantitative RT–PCR analysis of PGC‐1α expression in normal‐1 and HGPS‐1 fibroblasts treated with vehicle or 100 nM MB for 8 weeks (*P <* *0.05; **P <* *0.01). (E) Representative TEM graphs showing ultrastructure of the nuclear morphology at 3.15 k magnification (upper panel) and heterochromatin distribution along the nuclear envelope at 40 k magnification (lower panel) in HGPS‐1 fibroblasts treated with vehicle or MB for 8 weeks. (F) Representative immunofluorescence images of normal‐1 and HGPS‐1 fibroblasts treated with vehicle or MB 100 nm for 6 weeks. Green: anti‐HP1α antibody, Red: antilamin A/C antibody. Scale bar, 20 μm. (G) Western blotting analysis with anti‐HP1α or anti‐β‐actin antibodies in normal and two HGPS fibroblasts treated with vehicle or MB 100 nm for 6 weeks (upper panel). Normalized relative HP1α protein levels in each sample are shown in the lower graph.

To evaluate MB's effects on mitochondria, we compared ATP levels, mitochondrial superoxide (MitoSOX), and overall intracellular ROS (DCFDA) in mock‐ or MB‐treated cells. Consistent with MB's well‐documented antioxidant function, significant improvements in ATP production (Fig. 5A) and reductions in ROS levels (Fig. 5B) were found in MB‐treated cells compared to mock‐treated ones. Furthermore, TEM and fluorescence microscopy studies indicated a significant reduction in the number of severely defective mitochondria after MB treatment (Fig. 5C and Fig. S5D).

In addition, we found that MB treatment stimulated the expression of PGC‐1α (Fig. 5D) and thereby leading to a partial rescue of some of the PGC‐1α targeted mitochondrial genes (Fig. S5E). We concluded that the treatment with MB significantly improves mitochondrial functional and morphological abnormalities and stimulates PGC‐1α production. Notably, in contrast to the nuclear blebbing and progerin solubility analyses (Fig. 4) that appeared to be HGPS specific, the mitochondria in both normal and HGPS cells benefited from the MB treatment.

### MB rescues perinuclear heterochromatin loss and corrects misregulated gene expression in HGPS cells

Based on the results from Figs 4 and 5A–D, we hypothesize that while MB is a universal mitochondrial‐targeting antioxidant for both normal and HGPS cells, it has a specific role in HGPS cells by dislocating progerin away from the nuclear membrane. Previous studies reported that the anchorage of progerin to the nuclear membrane caused a loss of perinuclear heterochromatin in HGPS cells (Goldman *et al*., 2004; McCord *et al*., 2013). Using TEM technology, we examined this progerin‐directed nuclear phenotype in mock‐ and MB‐treated HGPS cells and observed an obvious restoration of perinuclear heterochromatin organization after MB treatment (Fig. 5E). The rescue of heterochromatin loss by MB was further verified by immunostaining and Western blotting using an antibody against heterochromatin protein 1‐alpha (HP1‐α). As a heterochromatic marker, HP1‐α was shown to be significantly downregulated in HGPS nuclei (Scaffidi & Misteli, 2005). Our experiments demonstrated that MB treatment led to an increase in nuclear HP1‐α staining (Fig. 5F) and an upregulation of HP1α protein level in HGPS cells (Fig. 5G), supporting our hypothesis.

**Figure 6:**
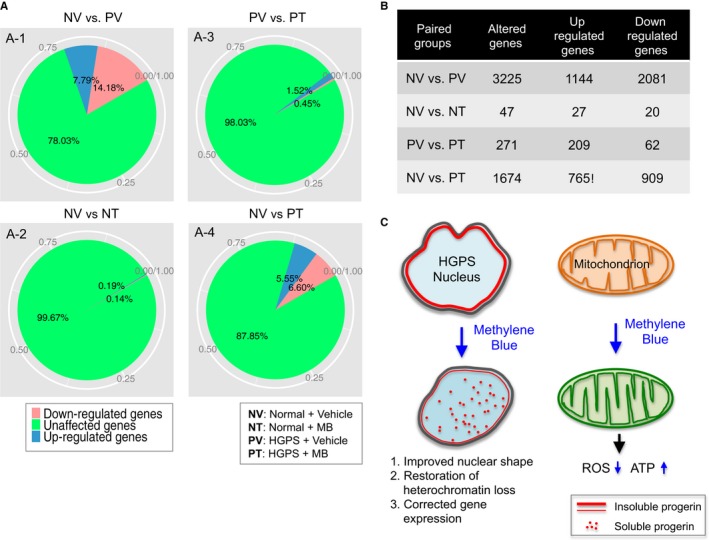
Methylene blue rescues gene expression in HGPS cells. (A) Pie chart showing the percentages and (B) the counted gene numbers of unaffected and affected genes in paired comparison among four groups (normal + vehicle vs. progeria + vehicle; normal + vehicle vs. normal + MB; progeria + vehicle vs. progeria + MB; normal + vehicle vs. progeria + MB). (C) Schematic summary of beneficial mechanisms of MB in HGPS cells. We find that MB treatment not only rescues mitochondrial defects (right) but also alleviates the HGPS hallmark nuclear blebbing, improves HGPS heterochromatin organization and corrects the expression of misregulated genes in the HGPS nucleus (left). The specific nuclear effects of MB on HGPS cells might be caused by the dislocation of progerin (solid red line) from the nuclear membrane to the nucleoplasm (red dots).

Chromatin re‐organization after MB treatment may induce gene expression changes. To examine MB's effects on gene expression, we conducted RNA‐seq in mock‐ and MB‐treated normal and HGPS cells and performed gene expression differential analysis using Tophat and Cufflinks suite of tools (Materials and methods). The RNA‐seq data were obtained from two biological replicates; each included four groups of samples (normal + vehicle; normal + MB; HGPS + vehicle and HGPS + MB). Pairwise comparisons were conducted (Fig. 6A,B). Consistent with previous studies (Ly *et al*., 2000; Park *et al*., 2001; Csoka *et al*., 2004; McCord *et al*., 2013), there are more than 20% differentially expressed genes (up/down: 7.79%/14.18%) in HGPS cells vs. normal fibroblasts (Fig. 6A‐1). We found these differentially expressed genes overlapped significantly with previous microarray and RNA‐seq studies (Cao *et al*., 2011a; McCord *et al*., 2013). When normal + vehicle vs. normal + MB was compared, only a few genes showed significant expression changes (up/down: 0.19%/0.14%, Fig. 6A‐2), indicating that MB basically does not interfere with normal gene expression. However, when comparing HGPS + Vehicle vs. HGPS + MB, we found a significant increase in genes affected by MB in the HGPS samples (up/down: 1.52%/0.45%, Fig. 6A‐3), which suggests that MB's effect on gene expression is relatively specific to HGPS cells, which might be related to the prior observation that MB specifically improved the solubility of progerin in HGPS cells, and had little effects on the solubility of lamin A/C in normal control. Significantly, after a 1‐month MB treatment, when comparing normal + vehicle vs. HGPS + MB, we found that the number of differentially expressed genes in MB‐treated HGPS cells was reduced to 1674 (Fig. 6A‐4), from 3225 in Fig. 6A‐1 (also see Fig. 6B). Collectively, the results demonstrated that MB specifically plays a role in the HGPS nucleus, restoring the perinuclear heterochromatin and correcting misregulated gene expression.

## Discussion

ROS can oxidize DNA, proteins, and lipids. It is agreed that cumulative oxidative damages by ROS is one of the causes of normal aging (Shigenaga *et al*., 1994). The majority of ROS is generated inside of mitochondria, and dysfunctional mitochondria will directly and indirectly elevate ROS production (Lin & Beal, 2006; Chen *et al*., 2007). Previous studies identified increased levels of ROS, reduced levels of mitochondrial proteins, and mitochondrial dysfunction in HGPS (Viteri *et al*., 2010; Lattanzi *et al*., 2012; Rivera‐Torres *et al*., 2013; Villa‐Bellosta *et al*., 2013). Our study continues to explore the connection between mitochondrial defects and HGPS‐associated premature aging. We demonstrate a significant enrichment of swollen and fragmented mitochondria and a marked reduction in mitochondrial movement in HGPS fibroblast cells (Figs 1 and 2A–C). Consistent with the morphological abnormalities, we detect a decline in various mitochondrial functional parameters in HGPS cells (Fig. 2D–G), including ATP production. All the described mitochondrial phenotypes hold true for both HGPS‐1 and HGPS‐2 cells. Interestingly, consistent with the progerin‐level differences, the HGPS‐2 always shows much severer defects than HGPS‐1. As the distribution and speed of mitochondrial traveling are closely associated with the cytoskeleton network and motors, our observation of the mitochondrial motility defect maybe also connected with the disruption of microtubule‐related proteins and the reduced availability of cellular ATP in HGPS cells (Rivera‐Torres *et al*., 2013 and Fig. 2G). To our knowledge, this is the first study that characterizes mitochondrial morphological and behavior defects in HGPS cells.

PGC‐1α is a transcriptional coactivator and a central inducer of mitochondrial biogenesis (Wu *et al*., 1999; Austin & St‐Pierre, 2012). Recent works have highlighted the role of PGC‐1α in balancing ROS in neurodegenerative disorders and aging by controlling both the induction of mitochondrial metabolism and the removal of its ROS by‐products (Austin & St‐Pierre, 2012). Our previous study on adipocyte differentiation has identified PGC‐1α as a major target that is inhibited by progerin during adipogenesis (Xiong *et al*., 2013). Consistent with those results in HGPS adipocytes, we observe a clear reduction of PGC‐1α protein in HGPS fibroblasts (Fig. 3A–C). Lentiviral induction experiment of GFP‐progerin in normal fibroblasts further indicates a causal and robust inhibitory effect of progerin on PGC‐1α's expression (Fig. 3E). Based on these observations, we initially speculated that an upregulation of PGC‐1α might potentially minimize the impact of ROS on cell physiology in HGPS. However, we find that overexpression of PGC‐1α in HGPS cells does not significantly improve mitochondrial phenotypes nor does it reduce ROS in HGPS cells (Fig. 3F,G). A possible explanation is that PGC‐1α is one of the many key metabolic genes affected by progerin expression, and thus, regulating this single factor may not be sufficient to rescue HGPS. Genomewide RNA‐seq and differential expression analysis supported our reasoning that the expression of over 3000 genes is altered in HGPS cells (Fig. 6A‐1).

In an attempt to rescue the mitochondrial defects, we examine the effects of MB, a potent antioxidant known to stimulate mitochondria, on HGPS fibroblasts. We find that MB treatment not only rescues mitochondrial defects (Fig. 5A–D) but also alleviates the HGPS hallmark nuclear blebbing phenotype (Fig. 4B,C). In addition, we find that MB treatment improves HGPS heterochromatin organization and corrects the expression of more than 50% of the misregulated genes in the HGPS nucleus (Figs 5E–G and 6). As a highly permeable molecule, it is known that MB can potentially enter all the other cellular compartments, including the nucleus (Kelner *et al*., 1988; Atamna & Kumar, 2010; Oz *et al*., 2011). Thus, the results in the nucleus were encouraging but not completely unexpected. What's surprising to us was that MB's impacts on the nucleus appear to be largely specific to HGPS (Figs 4B–J and 6), in contrast to MB's antioxidant effects on mitochondria that were comparable to both normal and HGPS (Fig. 5A–D). The first hint of this specificity was obtained from the microscopic analyses (Figs S5C and S6), where we noticed that progerin in MB‐treated HGPS cells was more solubilized and concentrated in nucleoplasm than that in mock treatment. Later, the biochemical fractionation experiments provided an independent line of evidence that MB treatment specifically enhances the solubility of progerin and thereby other A‐type lamins in HGPS cells, but has little effect on lamin A/C in normal cells (Fig. 4F–J). Previous literatures reported that releasing progerin from the nuclear membrane by farnesyltransferase inhibitor treatment partially rescued the gene expression and morphological defects in HGPS cells (Capell *et al*., 2005, 2008; Capell & Collins, 2006; Gelb *et al*., 2006; Cao *et al*., 2007; Wang *et al*., 2007; Marji *et al*., 2010). Indeed, we find that not only the nuclear morphology but also the chromatin organization and gene expression are improved in the MB‐treated HGPS nucleus (Fig. 6C). How MB solubilizes progerin remains unclear and will require further analysis. Future work will also be necessary to determine whether the reduction of differentially expressed genes after MB treatment have any beneficial effects as some overexpressed genes could play a compensatory role in diseases.

In summary, we reported mitochondrial morphological and behavior defects in HGPS cells and suggested a significant role of the mitochondrial dysfunction in the premature senescence phenotype development. Furthermore, this study suggests MB as a promising drug for HGPS, which remediates not only the damaged mitochondria but also the troubled nuclei in HGPS cells. Future study needs to be extended to determine the *in vivo* effects of MB in various HGPS mouse models.

## Materials and methods

### Cell culture and drug treatment

The normal and HGPS human skin fibroblast lines were obtained from Progeria Research Foundation (PRF) (detailed information described in Table S1). Both progeria cell lines carry the classic C1824T mutation. All fibroblast cell lines were cultured in MEM (Life Technologies, Carlsbad, California, United States) supplemented with 15% FBS (Gemini Bio‐Products, West Sacramento, CA, USA) and 2 mm l‐glutamine (Life Technologies) at 37 °C with 5% CO_2_. Methylene blue (MB; Acros Organics) was dissolved in PBS and added to the growth medium at a final concentration of 10 or 100 nm. N‐Acetyl‐L‐cysteine (NAC; Acros Organics) was dissolved in PBS and added to the growth medium at a final concentration of 1 mm. Fresh medium was provided twice a week, and the cultures were passaged 1:3 at 95% confluency.

### Generation of lentiviruses

Lentiviral constructs expressing GFP‐lamin A, GFP‐progerin, or PGC‐1α‐his (Addgene #10974) were made as previously described (Kageyama *et al*., 2014). In brief, the GFP‐lamin A, GFP‐progerin, or PGC‐1α‐his was subcloned into the pHR‐SIN‐CSGW vector using BamHI and NotI cloning sites. After verification by sequencing, these lentiviral constructs were cotransfected into HEK293T cells with two packaging vectors, pHR‐CMV‐8.2ΔR, and pCMV‐VSVG using Fugene 6 (Promega, E2692, Madison, WI, USA). Two days after transfection, the culture supernatant containing viruses was clarified by filtration through 0.45‐μm filters and stored at −80 °C.

### Transmission electron microscopy (TEM)

Passage‐matched normal or HGPS skin fibroblasts were grown on 35‐mm glass‐bottom dish until 80% confluence. Cells were prepared for transmission electron microscopy analysis following the protocol described previously (McCord *et al*., 2013). Briefly, cells were fixed with 2.5% glutaraldehyde in 0.1 m sodium cacodylate (pH 7.4) for 1 h at room temperature. Specimens were washed three times with 0.1 m sodium cacodylate and postfixed for 1 h with 1% osmium tetroxide in 0.1 m sodium cacodylate at room temperature. After washing three times with distilled water, specimens were incubated with 2% aqueous uranyl acetate at room temperature. The specimens were dehydrated in ethanol gradients: 35% for 10 min, 50% for 10 min, 70% for 10 min, 95% for 10 min, and 100% for 3 × 5 min. The infiltration was performed in EtOH: EPON resin mixture at various ratios, 1:1, 1:2, 1:3, or complete EPON for 1 h each ratio. The specimens were finally embedded in complete EPON at 60 °C overnight. A small piece of resin (2 × 2 mm) was cut to get ultra‐thin sections (70 nm) and the poststaining was performed in uranyl acetate, 2% aqueous for 5 min and lead citrate, 0.2–0.4% aqueous, for 1.5 min. Observation and micrographs were made with Zeiss EM10 CA. Mitochondrial morphological quantification was conducted blindly by a trained TEM expert. Two observers scored over 360 mitochondria from 10 randomly selected cells in each group blindly. Three general categories for phenotype grading were classified based on the intactness of membrane (outer, inner and cristae), matrix integrity, and overall organelle shape: mitochondria with intact membrane and matrix are considered as ‘normal’ (a‐b); mitochondria with broken membrane or with small vacuole areas in matrix (<20% of the total area) are considered as ‘minor defects’ (c‐d); and mitochondria that are either morphologically abnormal (swollen or budding) or with large vacuole areas (over 20% of the total area) are defined as ‘severe defects’ (e‐h). Over 300 mitochondria in either normal or HGPS lines were blindly scored according to these criteria.

### Mitochondrial live imaging

Skin fibroblasts were grown on a 35‐mm glass‐bottom dish until 60% confluence. For G1 phase synchronization, fibroblast cells were synchronized by serum starvation for 24 h. Mitochondria were stained with 50 nm of MitoTracker Green FM (M7514; Life Science) or 100 nm of MitoTracker Red CMXRos (M7512; Life Science) for 20 min then maintained in fresh culture medium at 37 °C, and 5% CO_2_ during imaging. The Mitochondrial live images were acquired using Velocity Suite (PerkinElmer, Hanover, MD, USA) on a spinning disk confocal microscopy system (UltraVIEW VoX; PerkinElmer) attached to an inverted microscope (Eclipse Ti; Nikon) with a 40 × 1.4 N.A. objective and equipped with a C9100‐50 camera (Hamamatsu). Images were acquired at an interval of 10 s for 5 min, and data analysis was performed using Velocity (version 6.3; PerkinElmer).

### Flow cytometry analysis for ROS, MMP, and apoptosis

To measure mitochondrial superoxide, cells cultured on 60‐mm dishes were incubated with fresh complete medium containing 5 μm MitoSOX Red (Life Technologies, M36008) at 37 °C. After 30 min, stained cells were harvested by trypsin digestion and rinsed twice with PBS. Single‐cell suspensions in 400 μL PBS were prepared for FACS analysis (FACS Canto II; BD). MitoSOX Red was excited by a laser at 488 nm, and the data were collected at 582 ± 21 nm. For cellular ROS measurement, cells grown on 60‐mm dishes were dissociated by trypsin digestion, rinsed with PBS, and then incubated in 1× dilution buffer containing 12.5 μm DCFDA (Abcam, Cambridge, MA, USA, ab113851) at 37 °C. After 30 min, the DCFDA was excited by laser at 488 nm, and the data were collected at 530 ± 15 nm. Mitochondrial membrane potential (MMP, Life Technologies, Carlsbad, CA, USA, M34152) and Annexin V‐positive apoptotic cells (BD Pharmingen, San Jose, CA, USA, 556547) were measured according to manufacturer's protocol. Flow cytometry was performed by FACS CantoII (BD), and the data were analyzed by FlowJo Ashland, OR, USA, software.

### RNA extraction, cDNA synthesis, and quantitative RT–PCR

Total RNA from various cell lines was extracted with Trizol (Life Technologies, Carlsbad, California, United States) and purified using the RNeasy Mini kit (Qiagen, USA) according to the manufacturer's instructions. The RNA yield was determined using the NanoDrop 2000 spectrophotometer. One microgram of total RNA was converted to cDNA using iScript Select cDNA Synthesis kit (Bio‐Rad, USA). Quantitative RT–PCR was performed in triplicate using SYBR Green Supermix (Bio‐Rad) on CFX96 real‐time system (C1000 Thermal Cycler; Bio‐Rad). All primers used in this study are listed in Table S2 (Supporting information).

For RNA sequencing experiment, the cell pellets of normal or HGPS fibroblasts were collected from two individually cultured cells treated with vehicle or 100 nm MB from P15 or P16 to P20. Total RNA was extracted with Trizol (Life Sciences), followed by RNA precipitation with isopropanol, then purified using the RNeasy Mini Kit (Qiagen). The RNA quality was checked using Agilent 2100 Bioanalyzer showing good RIN numbers (8.90–9.70) in all eight samples. The RNA‐seq sample preparation and sequencing were conducted according to the Illumina Truseq RNA sample preparation V2 guide by the IBBR Sequencing Core facility at the University of Maryland. RNA‐seq mapping and gene expression differential analysis was performed using Tophat and Cufflinks suite of tools as previously described (Trapnell *et al*. 2010, Trapnell *et al*. 2012; Trapnell, Pachter, and Salzberg 2009).

### Immunocytochemistry

Immunostaining was carried out using the following antibodies: lamin A/C (MAB3211; Millipore, Gibbstown, NJ, USA), progerin (Cao *et al*., 2011b), PGC‐1α (Thermo Scientific, USA), and DAPI (Vector Laboratories, Burlingame, CA, USA) was used to counterstain cell nuclei. Images were acquired with either Zeiss AX10 microscope equipped with a SPOT PURSUIT camera or Zeiss, USA LSM 710 confocal microscope. Fluorescence intensity was analyzed with ImageJ or a custom program (Driscoll *et al*., 2012).

### Western blotting

Whole cell lysates for immunoblotting were prepared by dissolving cells in Laemmli Sample Buffer containing 5% 2‐mercaptoethanol (Bio‐Rad). Antibodies used in this study included the following: PGC‐1α (KP9803; EMD, Millipore, USA), lamin A/C (sc‐6215; Santa Cruz), progerin (Cao *et al*., 2011b), HP‐1α (#2616; Cell Signaling, Danvers, MA, USA), p16 (sc‐468; Santa Cruz, Dallas, TX, USA), and β‐actin (A3854; Sigma‐Aldrich, USA).

### Senescence‐associated β‐galactosidase activity assay

SA–β‐gal activity assay was performed according to the manufacturer's protocol (#9860; Cell Signaling). Briefly, fibroblast cells grown on six‐well plate were fixed in 1X fixative solution containing 2% formaldehyde and 2% glutaraldehyde for 10 min and then stained overnight at 37 °C with the β‐galactosidase staining solution at pH 6.0 for 15 h. Images were acquired by Zeiss AX10 microscope with a SPOT PURSUIT camera.

### Fractionation of fibroblast nuclei

Fibroblast cells grown on a 100‐mm dish were harvested with 0.05% trypsin‐EDTA when they reached 70% confluence and rinsed with ice‐cold PBS twice. Nuclei were separated from cytoplasm following the manual of NE‐PER Nuclear and Cytoplasmic Extraction Reagents (#78835; Thermo Scientific). After centrifuging, the cytoplasm supernatant was removed. The pellets containing nuclei were resuspended well in lysis buffer (50 mm Tris–HCl, pH 7.4, 150 mm NaCl, 1% Triton X‐100, 0.1% deoxycholate, complete mini protease inhibitor cocktail tablet) and subjected to slight sonication at 20% amplitude for 30 s. (FB120; Fisher Scientific). The whole nuclei lysate was further centrifuged at 16 000 *g* for 5 min at cold. The supernatant was saved as the soluble fraction of the nuclei while the pellet was saved as the insoluble fraction of the nuclei. Both fractions of nuclei were prepared for Western blot assay by adding Laemmli sample buffer (Bio‐Rad). A one‐fifth portion of either soluble or insoluble fraction sample was loaded onto 10% SDS–PAGE gel and then proceeded for Western blot analysis. Images were taken with ChemiDoc^™^ Touch Imaging System (Bio‐Rad), and band intensity analysis was carried out with Image Lab software 5.2.1 (Bio‐Rad).

### ATP assay

Intracellular ATP content was measured using luminescence ATP detection system (ATPlite, PerkinElmer). Briefly, fibroblast cells were harvested with 0.05% trypsin‐EDTA and counted. The same number of 2500 cells from each fibroblast sample was seeded onto a 96‐well black plate (triplicate). After the cells had been lysed with the lysis buffer for 5 min, the substrate solution was added and mixed for another 5 min to conduct the reaction for light generation. After dark adaption for 10 min, the luminescence intensity of each well was acquired at luminescence mode with SoftMax Pro software connecting to SpectraMax M5 Microplate Reader.

### Quantification of mitochondrial DNA (mtDNA)

The whole DNA including genomic and mitochondrial DNA from fibroblasts was extracted with UltraPure^™^ Phenol: Chloroform: Isoamyl Alcohol (25:24:1) (15593‐031, ThermoFisher Scientific, USA). Instead of proceeding to the column isolation, DNA was precipitated with ethanol to avoid mtDNA loss. DNA concentration was measured using a NanoDrop 2000 spectrophotometer (Thermo Scientific). The total amount of 100 ng of DNA was added into a 15‐μL qPCR system with either mtDNA primers or s18 RNA primers (Table S2). The level of mtDNA was calculated using the delta *C*_t_ (Δ*C*_t_) of average *C*_t_ of mtDNA and nDNA (Δ*C*_t_ = CtmtDNA‐CtnDNA) in the same well as an exponent of 2 (2Δ*C*_t_).

### Statistical analysis

Results are presented as the mean ± standard deviation. Data were analyzed using 2‐tailed Student's *t*‐test, and a *P* value < 0.05 was considered significant. A chi‐squared test was conducted to compare the distribution difference of mitochondria with various ultrastructural abnormalities in normal and HGPS fibroblasts.

## Funding

This work was supported by R21AG043801 (KC).

## Conflict of interest

None declared.

## Supporting information


**Fig. S1** (A) Western blot analysis with antiprogerin or anti‐β‐actin antibodies in two normal and two HGPS fibroblasts (upper panel) and the corresponding progerin band intensity analysis normalized to β‐actin loading control (lower panel). (B,C) Cell cycle analysis with propidium iodide (PI) in normal‐1 and HGPS‐1 fibroblasts (B) and the corresponding percentages of each cell phase (C). (D) Representative fluorescence images of mitochondria stained with MitoTracker Red CMXRos at G1 phase of normal‐1 or HGPS‐1 fibroblast. Scale bar, 20 μm.
**Fig. S2** Defective mitochondrial mobility in progerin‐expressing cells.
**Fig. S3** Flow cytometry profiles of MMP and PI/Annexin apoptosis analysis.
**Fig. S4** Mitochondrial defects in HGPS iSMCs.
**Fig. S5** Methylene blue delays cellular senescence and improves mitochondrial defects in HGPS fibroblasts.
**Fig. S6** Methylene blue increases nucleoplasmic progerin in HGPS fibroblasts.
**Table S1** Fibroblast cell line information.
**Table S2** Primer sequences.

**Table S3** Differentially expressed gene list.
