# Supplementary material for: Methylene blue alleviates nuclear and mitochondrial abnormalities in progeria
Source: Aging Cell. 2015 Dec 14;15(2):279–90. doi: 10.1111/acel.12434 (PMC4783354; doi:10.1111/acel.12434)
Supplement: Supplementary file 1 — Fig. S1 (A) Western blot analysis with antiprogerin or anti‐β‐actin antibodies in two normal and two HGPS fibroblasts (upper panel) and the corresponding progerin band intensity analysis normalized to β‐actin loading control (lower panel). (B,C) Cell cycle analysis with propidium iodide (PI) in normal‐1 and HGPS‐1 fibroblasts (B) and the corresponding percentages of each cell phase (C). (D) Representative fluorescence images of mitochondria stained with MitoTracker Red CMXRos at G1 phase of normal‐1 or HGPS‐1 fibroblast. Scale bar, 20 μm. Fig. S2 Defective mitochondrial mobility in progerin‐expressing cells. Fig. S3 Flow cytometry profiles of MMP and PI/Annexin apoptosis analysis. Fig. S4 Mitochondrial defects in HGPS iSMCs. Fig. S5 Methylene blue delays cellular senescence and improves mitochondrial defects in HGPS fibroblasts. Fig. S6 Methylene blue increases nucleoplasmic progerin in HGPS fibroblasts. Table S1 Fibroblast cell line information. Table S2 Primer sequences. [file ACEL-15-279-s001.pdf]

Supplemental Figure 1

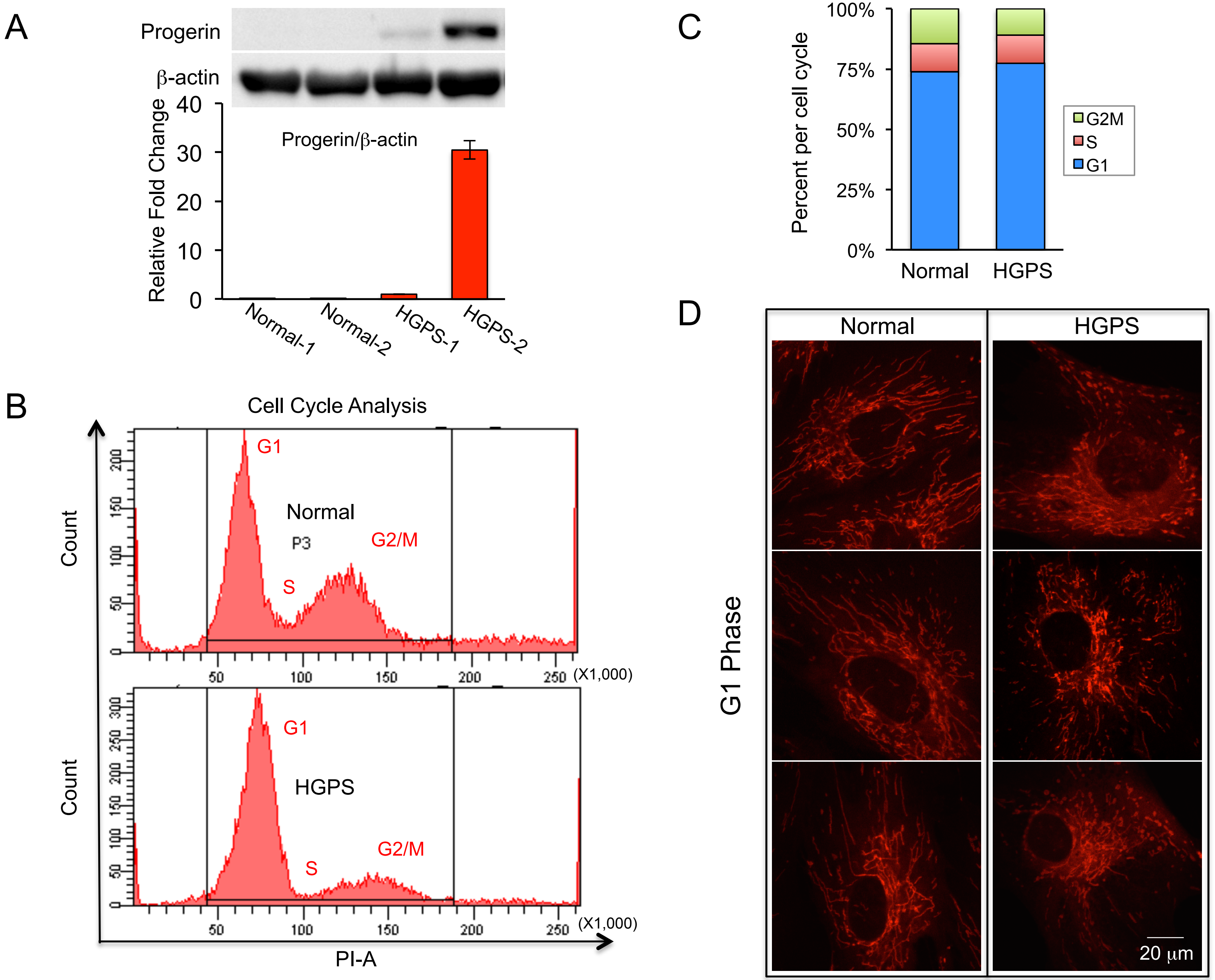

Supplemental Figure 2

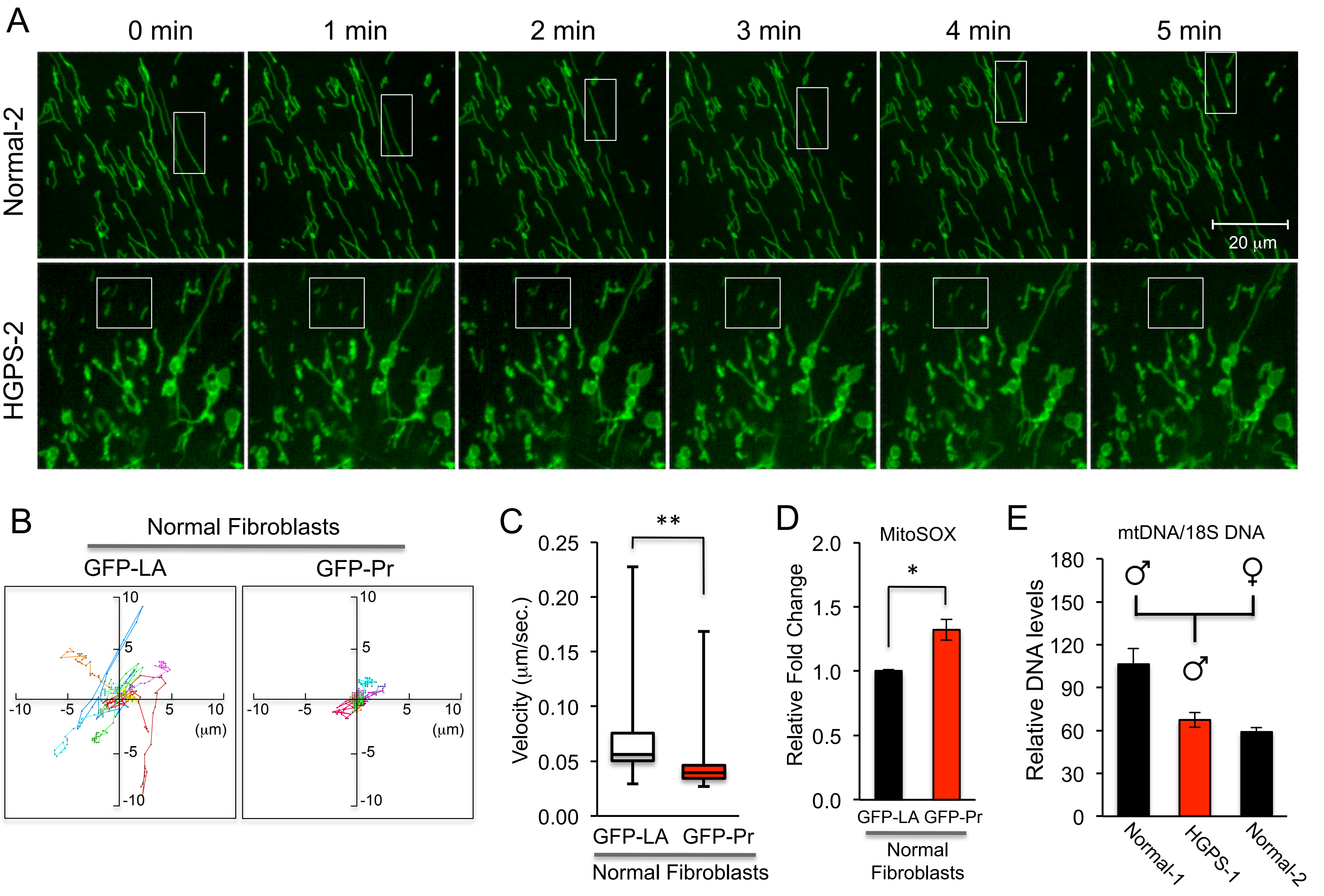

Supplemental Figure 3

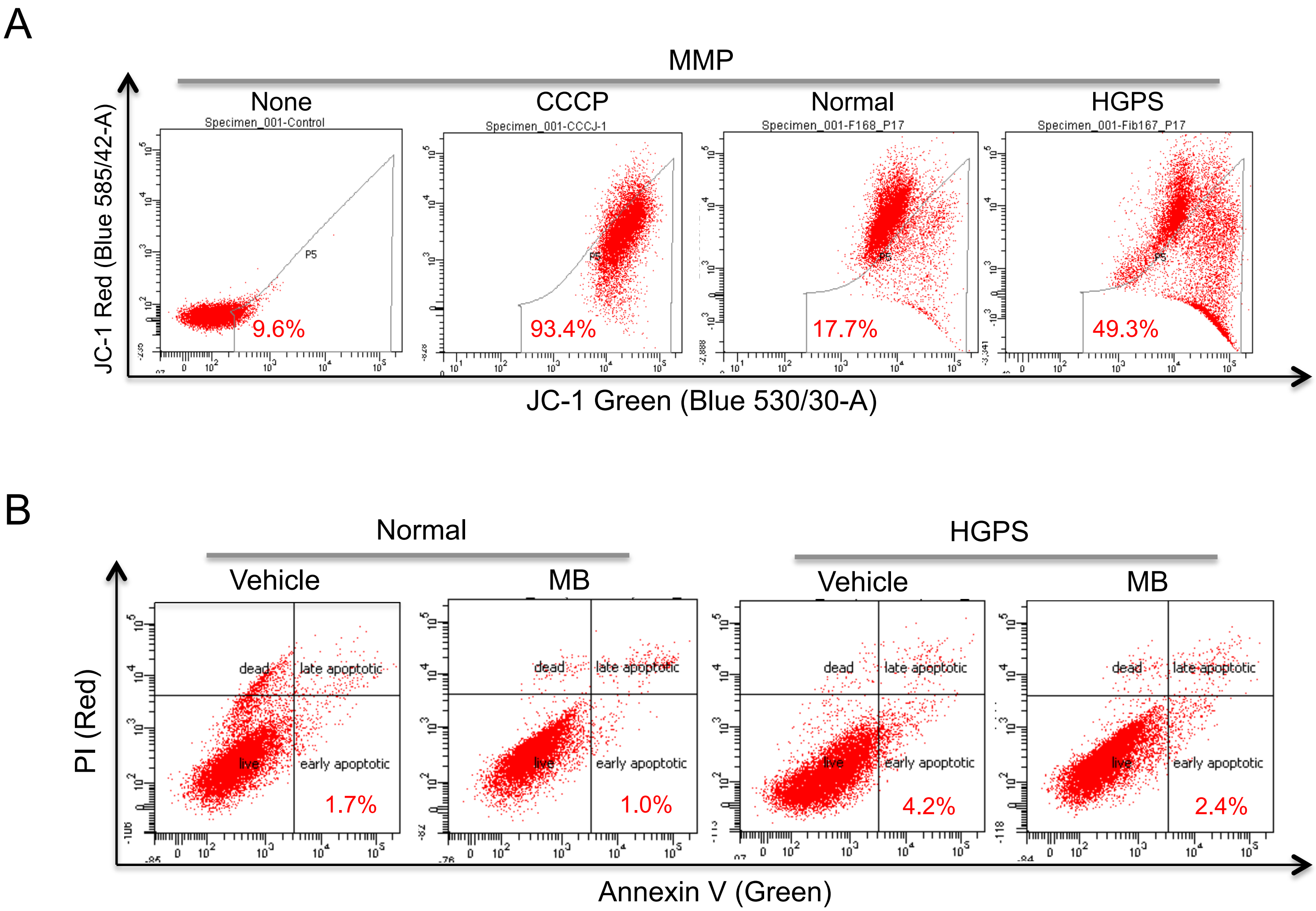

Supplemental Figure 4

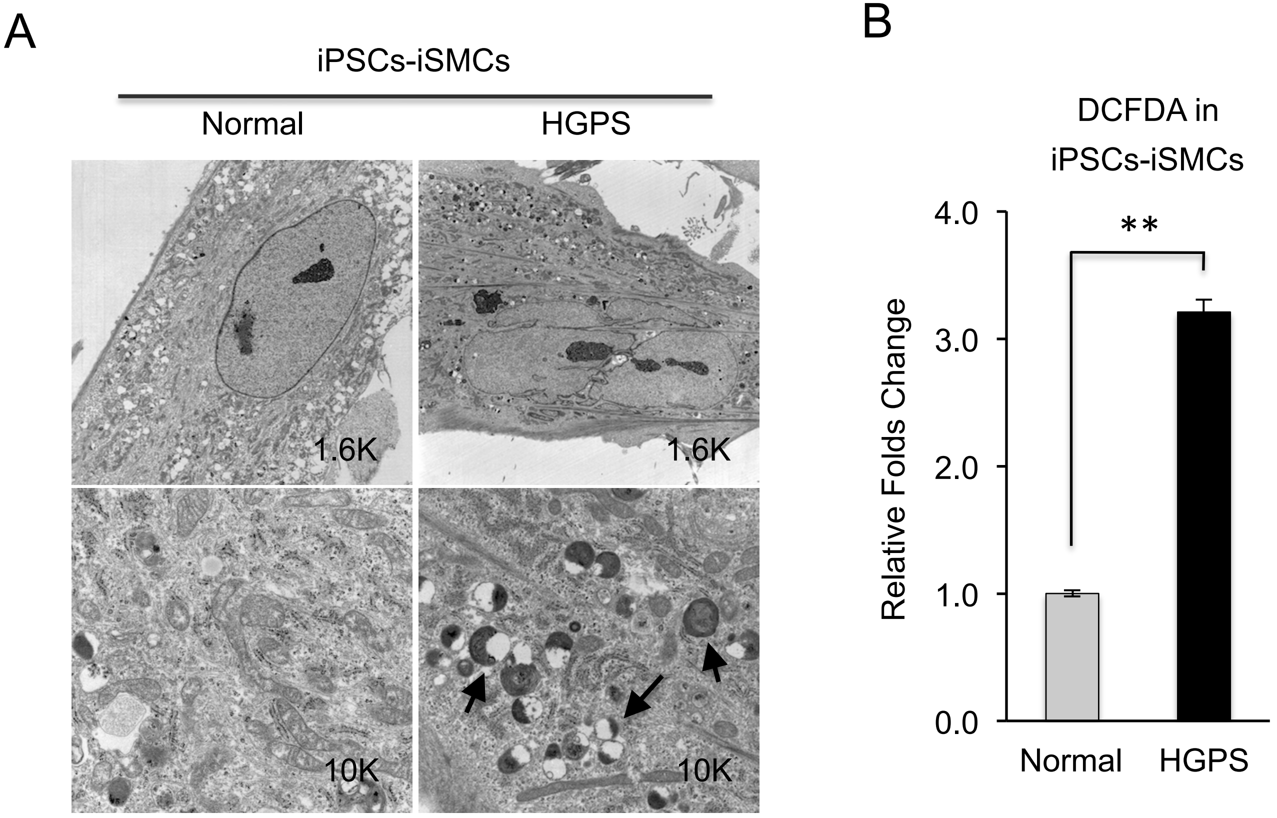

Supplemental Figure 5

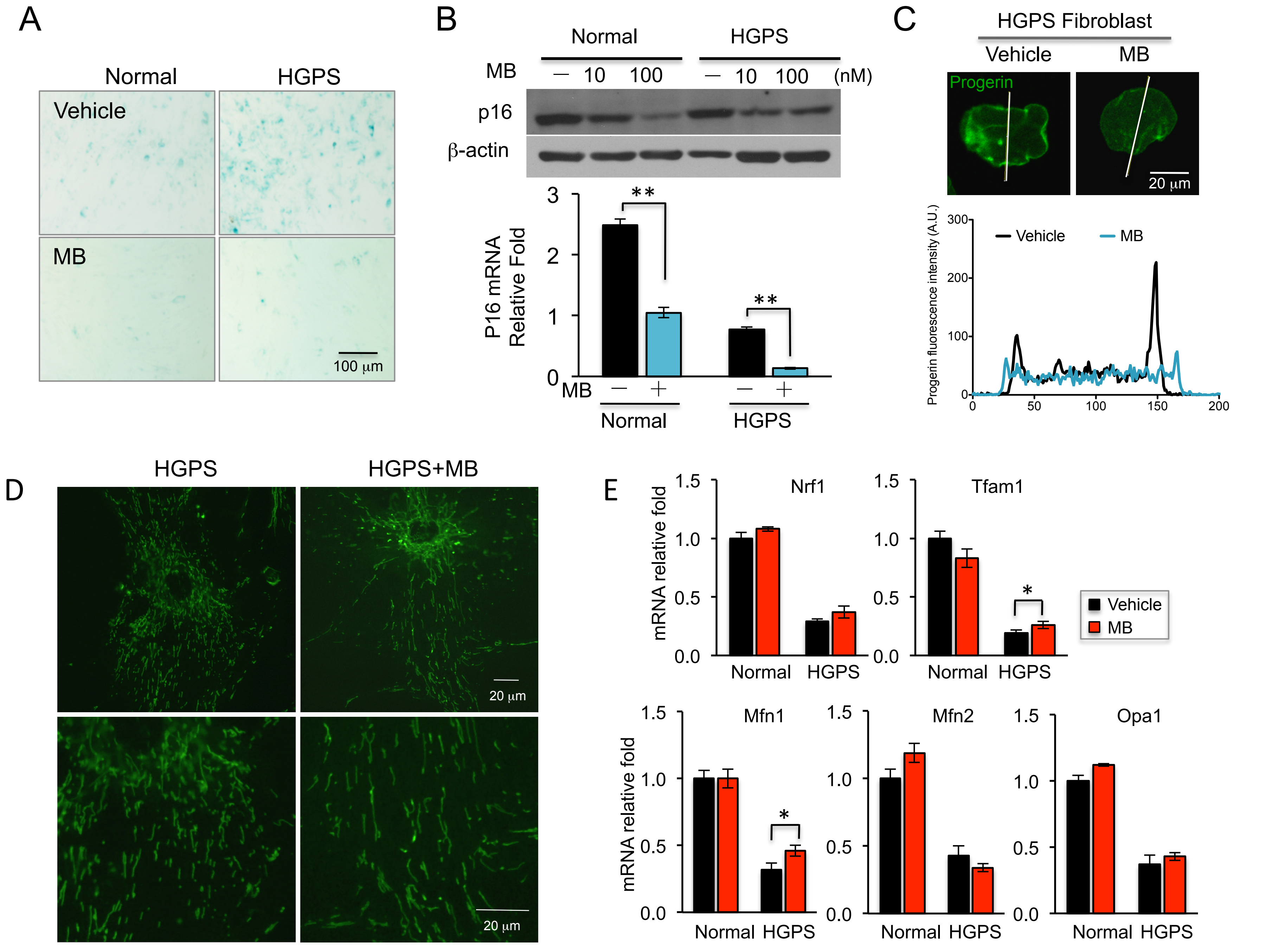

Supplemental Figure 6

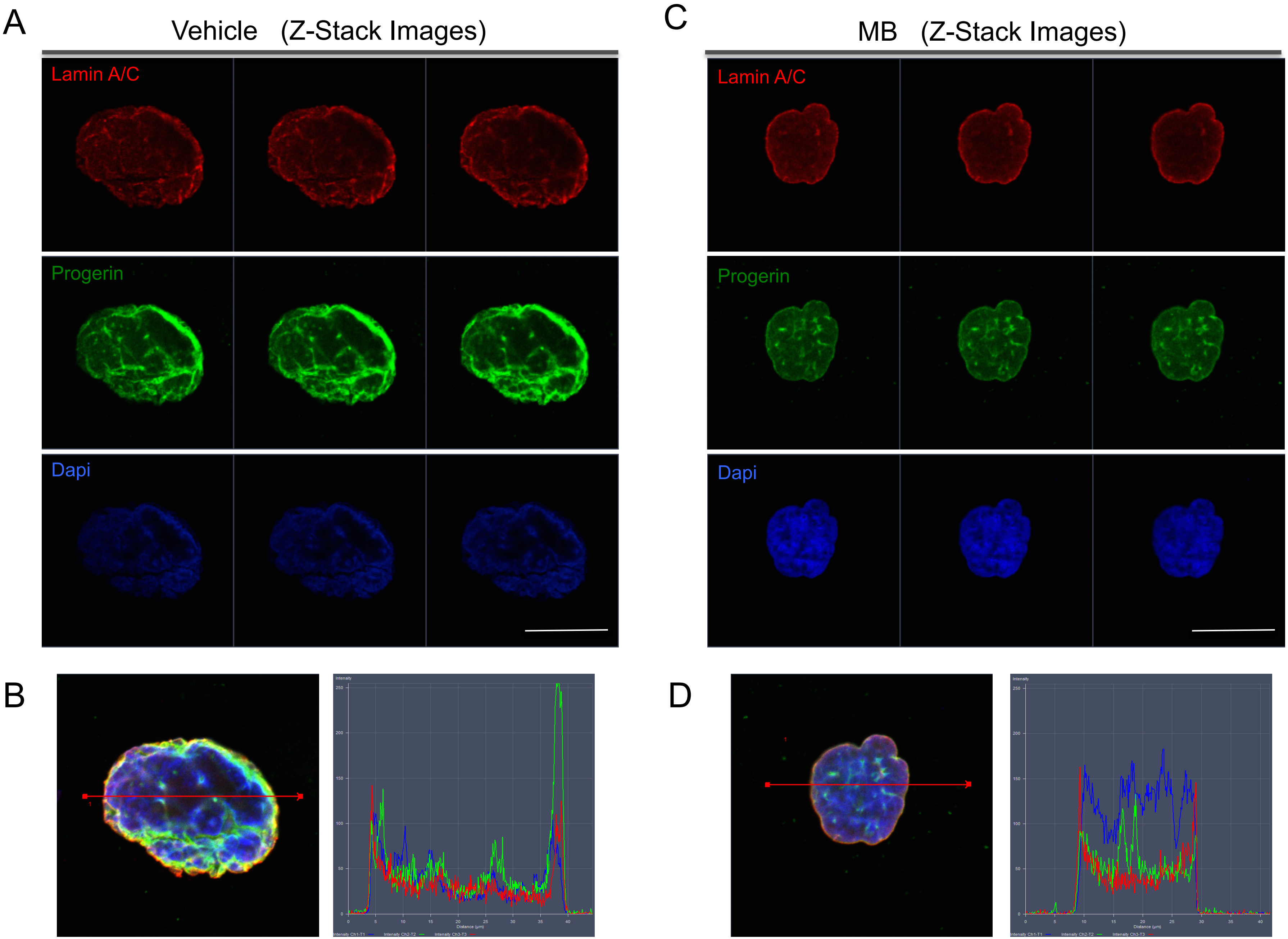

Supplemental Table 1. Fibroblast Cell Line information

Cell line (Name in paper)	Passage Number * (Starting → Ending)	Gender	Donor Age	Mutation	Resource	Other
HGFDFN168 (Normal-1 or Normal)	P10 → P22	Male	40 yrs 5 mos	None	PRF **	Father
HGMDFN090 (Normal-2)	P10 → P22	Female	37 yrs 10 mos	None	PRF	Mom
HGADFN167 (HGPS-1 or HGPS)	P10 → P22	Male	8 yrs 5 mos	G608G	PRF	Son
HGADFN169 (HGPS-2)	P10 → P17	Male	8 yrs 6 mos	G608G	PRF	Unrelated child

*Normal and HGPS cell lines were carefully matched for passage number in each experiment.

** PRF - Progeria Research Foundation

Supplemental Table 2. Primer sequences for quantitative PCR (Human)

Gene	Sequence	
Lamin A	Sense	5'-GCAACAAGTCCAATGAGGACCA-3'
	Antisense	5'-CATGATGCTGCAGTTCTGGGGGCTCTGGAT-3'
Progerin	Sense	5'-GCAACAAGTCCAATGAGGACCA-3'
	Antisense	5'-CATGATGCTGCAGTTCTGGGGGCTCTGGAC-3'
Lamin C	Sense	5'-CTGCGTACGGCTCTCATCA-3'
	Antisense	5'-CGGCGGCTACCACTCAC -3'
b-actin	Sense	5'-CTGGAACGGTGAAGGTGACA-3'
	Antisense	5'-AAGGGACTTCCTGTAACAATG CA-3'
PGC-1a	Sense	5'-TGCCCTGGATTG TTGACATGA-3'
	Antisense	5'-TTTGTCAGGCTGGGGGTAGG-3'
18S rRNA	Sense	5'-TAGAGGGACAAGTGGCGTTC-3'
	Antisense	5′-CGCTGAGCCAGTCAGTGT-3'
mtDNA	Sense	5'-CACCCAAGAACAGGGTTTGT-3'
	Antisense	5′-TGGCCATGGGTATGTTGTTAA-3'
Nrf1	Sense	5'-GGAGTGATGTCCGCACAGAA-3'
	Antisense	5'-CGCTGTTAA GCGCCATAGTG-3'
Tfam1	Sense	5'-GACTTCTGCCAGCATAATAC-3'
	Antisense	5'-GAGTTCTGCCTGCTTTATG-3'
Mfn1	Sense	5'-CTCCAGCAACGCCAGATAATGC-3'
	Antisense	5'-ACTTGTTGGCACAGGCGAGC-3'
Mfn2	Sense	5'-GGATGC TGATGTGTTTGTGCTGG-3'
	Antisense	5'-GGATGCTGATGTGTTTGTGCT GG-3'
Opa1	Sense	5'-GGCTCTGCAGGCTCGTCTCAAGG-3'
	Antisense	5'-TTCCGCCAGTTGAACGCGTTTACC-3'
p16	Sense	5′-TAGTTACGGTCGG AGGCCGAT
	Antisense	5′-CTTTCAATCGGGGATGTCTGAG
Fis1	Sense	5'-CGAGCTGGTGTCTGTGGAGGACC-3'
	Antisense	5'-TGTCAA TGAGCCGCTCCAGTTCC-3'
Drp1	Sense	5'-AACTTGATCTCATGGATGCGGG-3'
	Antisense	5'-ATGAACCAGTTCCACACAGCGG-3'
